# Regulating the Electron Distribution of Metal‐Oxygen for Enhanced Oxygen Stability in Li‐rich Layered Cathodes

**DOI:** 10.1002/advs.202307397

**Published:** 2024-04-22

**Authors:** Zijia Yin, Jun Zhao, Dong Luo, Yi‐Ying Chin, Chien‐Te Chen, Huaican Chen, Wen Yin, Yu Tang, Tingting Yang, Jincan Ren, Tianyi Li, Kamila M. Wiaderek, Qingyu Kong, Jun Fan, He Zhu, Yang Ren, Qi Liu

**Affiliations:** ^1^ Department of Physics City University of Hong Kong Hong Kong 999077 P. R. China; ^2^ Shenzhen Research Institute City University of Hong Kong Shenzhen Guangdong 518057 P. R. China; ^3^ Department of Materials Science and Engineering City University of Hong Kong Hong Kong 999077 P. R. China; ^4^ Department of Physics National Chung Cheng University No.168, Sec. 1, University Rd., Minhsiung Chiayi 621301 Taiwan; ^5^ National Synchrotron Radiation Research Center 101 Hsin‐Ann Road Hsinchu 30076 Taiwan; ^6^ Institute of High Energy Physics Chinese Academy of Sciences (CAS) Beijing 100049 P. R. China; ^7^ X‐Ray Science Division Argonne National Laboratory Lemont IL 60439 USA; ^8^ Société Civile Synchrotron SOLEIL L'Orme des Merisiers Saint‐Aubin, BP 48 GIF‐sur‐Yvette Cedex 91192 France; ^9^ Herbert Gleiter Institute of Nanoscience School of Materials Science and Engineering Nanjing University of Science and Technology Nanjing 210094 P. R. China

**Keywords:** delocalized electrons, electronic modulation, in situ characterization, lithium‐rich oxide cathodes, oxygen stability

## Abstract

Li‐rich Mn‐based layered oxides (LLO) hold great promise as cathode materials for lithium‐ion batteries (LIBs) due to their unique oxygen redox (OR) chemistry, which enables additional capacity. However, the LLOs face challenges related to the instability of their OR process due to the weak transition metal (TM)‐oxygen bond, leading to oxygen loss and irreversible phase transition that results in severe capacity and voltage decay. Herein, a synergistic electronic regulation strategy of surface and interior structures to enhance oxygen stability is proposed. In the interior of the materials, the local electrons around TM and O atoms may be delocalized by surrounding Mo atoms, facilitating the formation of stronger TM─O bonds at high voltages. Besides, on the surface, the highly reactive O atoms with lone pairs of electrons are passivated by additional TM atoms, which provides a more stable TM─O framework. Hence, this strategy stabilizes the oxygen and hinders TM migration, which enhances the reversibility in structural evolution, leading to increased capacity and voltage retention. This work presents an efficient approach to enhance the performance of LLOs through surface‐to‐interior electronic structure modulation, while also contributing to a deeper understanding of their redox reaction.

## Graphical abstract

Here a synergistic strategy in Li‐richMn‐based cathodes is proposed to enhance oxygen stability. In this way, lonepair electrons of surface oxygen are passivated, while the local electronsaround transition metal (TM) and O atoms in the bulk are delocalized by Moatoms, forming stronger TM‐O bonds under high voltages. Thus, the reversibilityof the structure is significantly improved.

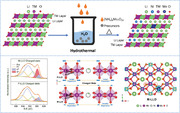

## 1 Introduction

Driven by the rapid development of the electric vehicle (EV) industry, there is an increasing global demand for high energy density and low‐cost rechargeable lithium‐ion batteries (LIBs).^[^ ^1^ ^]^ However, the development of LIBs is often hindered by the search for suitable cathode materials, as the anode electrodes typically possess relatively higher capacity and lower cost. Among the currently existing cathode materials, layered lithium‐rich Mn‐based oxide cathodes (LLOs) with high specific capacities (>250 mAh g^−1^) are regarded as the most promising candidates to alleviate the cost and constraints associated with EVs.^[^ ^2^ ^]^ The exceptional capacity provided by LLOs is attributed to the oxygen redox (OR) reaction that occurs at high voltages (> 4.5 V), in addition to the redox process of the cations.^[^ ^3^ ^]^ Unfortunately, several issues urgently need to be solved. In detail, at high voltage, the irreversible OR process brings about irreversible structural changes and oxygen release, exacerbating the transition metal (TM) migration and phase transition. These factors contribute to a continuous decrease in the capacity and voltage during cycling.^[^ ^4^ ^]^

Typically, the LLOs show a composite structure comprising a layered LiTMO_2_ and a monoclinic Li_2_MnO_3_ phase. The irreversible OR process is closely associated with the unstable oxygen framework within the Li_2_MnO_3_ phase at high voltages.^[^ ^5^ ^]^ Specifically, within the Li_2_MnO_3_ (Li[Li_1/3_Mn_2/3_]O_2_) phase, one‐third of Mn atoms in the TM slabs are orderly substituted by the Li atoms, resulting in the formation of a honeycomb structure.^[^ ^6^ ^]^ This honeycomb structure involves weak bonding between Li and adjacent oxygen atoms, which generates shallowed O lone pair electrons that participate in the OR process.^[^ ^7^ ^]^ However, at high voltage, the generated O‐holes from the O lone‐pair electrons extraction process also induce a shrinkage of O─O distance, which promotes O─O dimers formation and subsequent O_2_ release.^[^ ^8^ ^]^ So, stabilizing the honeycomb oxygen relies on the modulation of the electronic structure of O lone pairs.

To enhance oxygen stability, conventional methods include element doping,^[^ ^11^ ^]^ morphology regulation,^[^ ^12^ ^]^ surface coating,^[^ ^13^ ^]^ *etc*., with limited progress achieved, so more innovative ideas especially for lone‐pair electron modulation are still awaited to be proposed. Recently, theoretical investigations have highlighted the key role of TM─O covalency in stabilizing the O lone‐pair holes during the OR process and enhancing the oxygen stability in the Li‐rich materials.^[^ ^9^ ^]^ These studies proposed that the formed O─O dimers during the lone pair electrons extraction process can be stabilized by covalent bonding with the TM through TM(*d*)‐O_2_(*σ*) orbital interactions, preventing irreversible structural changes and oxygen release that induce reversible anionic redox process. However, achieving improved TM─O covalency at high voltages remains a significant challenge that has yet to be overcome.

In this work, we propose a strategy to regulate the electron distribution of metal‐oxygen for both interior and surface in LLO (i.e., Li_1.2_Ni_0.2_Mn_0.6_O_2_) materials, enhancing oxygen stability. On the surface of the modified LLO sample (i.e., M‐LLO), the highly active lone‐pair O 2p electrons will be coupled with the *d* electrons of extra TM atoms. This is beneficial to alleviate the irreversible vigorous OR process initiated by unsaturated oxygen atoms on the surface, which stabilizes the TM─O framework during charge‐discharge cycles and suppresses the O_2_ release.^[^ ^10^ ^]^ Meanwhile, in the interior of the M‐LLO sample, the electrons between Mn and O are delocalized by surrounding doping Mo atoms which enhances the Mn and O electron cloud overlapping, and forms short, strongly covalent Mn─O bonds at high voltages. Compared to the pristine sample (i.e., P‐LLO), the shorter TM─O bonds with a stronger bond energy of the charged M‐LLO sample can effectively stabilize the O atom and inhibit the formation of O─O dimers. By employing this electron regulation strategy for both surface and internal structures, reversible structural evolution and suppressed oxygen release of the M‐LLO are observed through in‐situ synchrotron X‐ray diffraction (XRD) and differential electrochemical mass spectrometry (DEMS). Consequently, the M‐LLO sample shows better capacity stability (95.10%) and lower voltage drop (224 mV) after 100 cycles.

## 2 Results and Discussion

### 2.1 Surface and Interior Structure

The M‐LLO cathode is designed and successfully prepared using a one‐step precursor hydrothermal treatment to achieve the Mo‐doping in the bulk phase and the enrichment of TMs on the surface simultaneously. The corresponding modifying process is illustrated in Figure S1 (Supporting Information). First, the precursor particles Ni_0.25_Mn_0.75_CO_3_ are hydrothermally treated in ammonium molybdate solution to obtain Mo‐modified precursors. After treatment, a large amount of Mo is concentrated on the surface of the particles. Then, during the molten salt‐assisted sintering process, some Mo atoms are doped into the Ni sites in bulk structure. While the surface Li─O─Li configuration of LLOs is leached by the Mo species at high temperature (Since Li─O─Li on the surface easily reacts with Mo species to form lithium molybdate, which has high solubility in water) and then during the washing process, the surface lithium molybdate is washed out to obtain a lithium‐poor surface structure material.^[^ ^14^ ^]^ The surface vacancies created by extracting Li atoms from the superlattice structure and the alkali metal layer will be occupied by Ni atoms replaced by Mo, forming a surface Ni‐enrichment structure.

**Figure 1:**
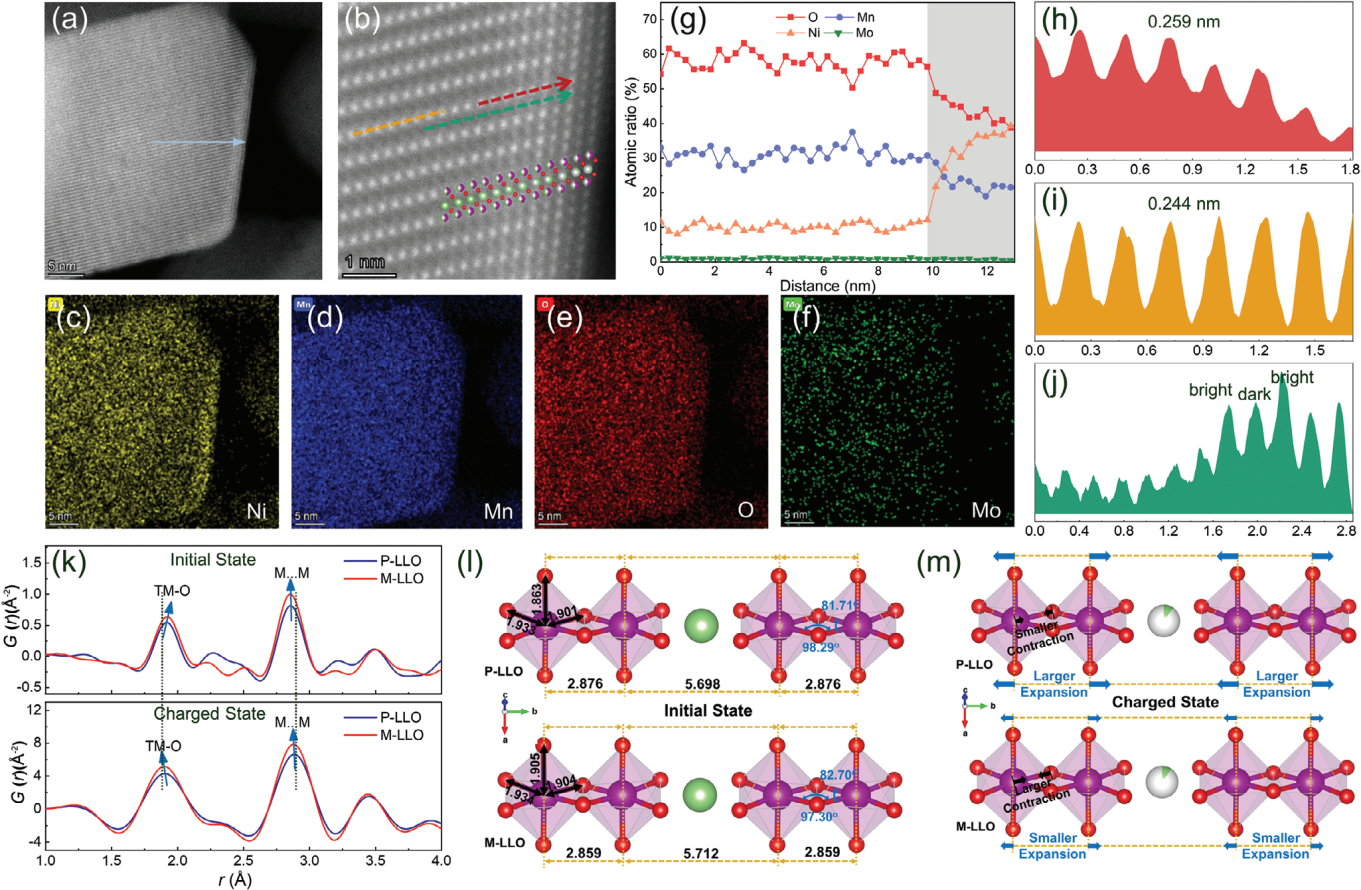
The crystal structure of the prepared samples. a) The HADDF‐STEM images of the M‐LLO sample. b) The enlarged area in 1a, emphasizes the atomic arrangement. c–f) STEM‐EDS mapping of the M‐LLO sample based on the selected region in 1a, which illustrates the element enrichment structure with enrichment of Ni at the surface. g) The curve of element distribution with the distance of the M‐LLO sample under STEM‐EDS scan mode based on the selected region in 1a along the blue line. h–j) The corresponding in‐line profile of the TM layer (orange line for bulk phase, red line for the surface) and the Li layer (green line). k) Low‐*r* PDF patterns of the P‐LLO and M‐LLO samples at the initial and charged states. l) Schematic illustration of the local structure for the P‐LLO and M‐LLO samples at the initial states. m) Schematic illustration of the local structure for the P‐LLO and M‐LLO samples at the charged states. The purple, green, and red spheres represent the TM, Li, and O atoms, respectively.

The extraction of the Li atoms from the superlattice structure and alkali metal layer promotes the surface enrichment of the heavier TM elements. The structure and the atomic arrangement from the bulk to the surface are further visualized by the atomic resolution high angle annular dark field scanning transmission electron microscopy (HAADF‐STEM) imaging. In the initial (**Figure** **1**a) and enlarged STEM images (Figure 1b), the layer structure and special surface structure are observed in the M‐LLO cathodes. To identify the element distribution and phase composition, X‐ray energy dispersive spectroscopy (EDS) mapping is employed. The results presented in Figure 1c–f illustrate that Mn, Mo, and O elements are evenly distributed in the entire particles, while the Ni element is not only uniformly distributed within the bulk phase but also enriched on the surface. Correspondingly, in Table S1 (Supporting Information), the atomic ratios of the Ni: Mn: Mo in STEM‐EDS is ≈10.4: 31.0: 0.9, which is close to the chemical composition of the inductively coupled plasma results. This implies that this selected area contains both Li_2_MnO_3_ and LiNi_0.5_Mn_0.5_O_2_ compositions. Meanwhile, the elemental distribution in the 13 nm depth of the surface (arrow in Figure 1a) is determined by line‐scanning mode (Figure 1g). The results show that the Ni atoms are distributed in a gradient approaching the chemical composition of the bulk phase at a depth of 3 nm, and the corresponding surface model is shown in the inset in Figure 1b. Besides, this Ni‐enrichment structure will cause the surface lattice spacing (Figure 1h, along the red arrow line in Figure 1b) to expand relative to the bulk region (Figure 1i, along the yellow arrow line). The Li layer (Figure 1j, along the green arrow line in Figure 1b) is partially occupied by TM atoms, and the surface bright‐dark‐bright lattice stripes imply that the Ni enrichment structure here is similar to the rock‐salt and spinel mixed phase.^[^ ^15^ ^]^

To illustrate the changes in the lattice structure and local ligand geometry, synchrotron XRD experiments are carried out. As shown in Figure S2 (Supporting Information), P‐LLO, and M‐LLO exhibit similar XRD patterns, and most of the diffraction peaks are well separated and indexed to a monoclinic (*C2/m* space group) Li_2_MnO_3_‐like structure with higher crystallinity and layered structure characteristics.^[^ ^16^ ^]^ The broad peaks in the region of 20–25° reflected the ordered superlattice arrangement of the Li and Mn atoms in the TM layer. The Rietveld refinement results (Table S2, Supporting Information) show subtle differences in the lattice structure with a slight expansion along the *a*/*c* axis in the M‐LLO cathode. To accurately detect the structural changes after the synergistic treatment, pair distribution function (PDF) analysis based on synchrotron total scattering data collection is employed. In particular, the PDF method has been proven to be effective in encoding the short‐range structural changes of the bond length and bond angle in the octahedron.^[^ ^17^ ^]^ Figure 1k shows the low‐*r* PDF patterns of the samples in the initial states (top subgraph) and charged states (bottom subgraph). In the initial state, the first peak ascribed to TM─O bonds in the M‐LLO is found to shift to the right, indicating that the TM─O bonds in M‐LLO are expanded compared with the P‐LLO.^[^ ^18^ ^]^ Corresponding refinement results and schematic representations of local octahedral configurations in Figure S4 (Supporting Information) and Figure 1l show that the TM─O bonds in M‐LLO expand from 1.863 to 1.905 Å compared with the P‐LLO. Meanwhile, in the M‐LLO cathode, the relative intensity of the second peak attributed to TM…Li or TM…TM scattering is enhanced, which is known to be caused by the decrease of the Li_2_MnO_3_ phase.^[^ ^18^ ^]^ It indicates that more Li sites on the surface are occupied by TM metals which leads to higher scattering intensity. The PDF spectra in the charged state show that the TM─O bond length is shortened and the M…M pair distance for both samples after charging is expanded, compared to that of the samples in the initial state. Moreover, in the charged state, the TM─O bond length and the M…M pair distance of the M‐LLO sample are slightly shorter than that of the P‐LLO sample. Therefore, as shown in the schematic diagram of Figure 1m, it implies that the M‐LLO sample exhibits a larger contraction of the TM─O bond and a smaller expansion of the M…M pair distance during charging compared to that of the P‐LLO sample.

Therefore, this Mo‐hydrothermal treatment method achieves the surface Ni‐enrichment and internal Mo‐doping structures simultaneously, which leads to the structural modification of the surface and the interior of LLO cathodes.

### 2.2 Electrochemical Performance

**Figure 2:**
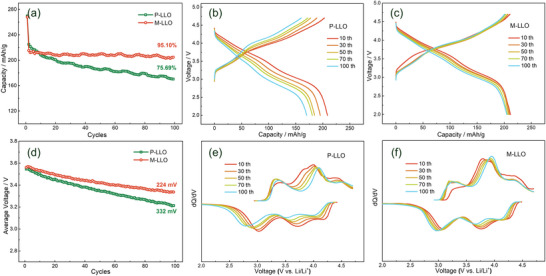
a) Cycling performance of P‐LLO and M‐LLO samples at 1 C between 2.0–4.7 V. Capacity‐voltage profiles of the b) P‐LLO and c) M‐LLO samples at 1C between 2.0–4.7 V. d) Average voltage performance of P‐LLO and M‐LLO samples at 1 C for 100 cycles. The corresponding *dQ/dV* curves of the e) P‐LLO and f) M‐LLO samples between 2.0–4.7 V for the 10th, 30th, 50th, 70th and 100th cycles.

The electrochemical properties of the P‐LLO and M‐LLO cathodes are evaluated between 2.0‐4.7 V after the initial activation charging/discharging process at 0.1 rate. The initial capacity‐voltage (CV) profiles of the two samples are displayed in Figure S4 (Supporting Information). For both samples, the first charging curves consist of two parts, a sloped curve, and a long plateau. The sloped curve (3.75‐4.4 V) is attributed to the TM oxidation with the removal of Li^+^ from the layered structure. The following long plateau (4.4‐4.7 V) corresponds to the activation process of the Li_2_MnO_3_ component with the extraction of Li_2_O from the Li_2_MnO_3_ phase.^[^ ^3^, ^19^ ^]^ After initial discharging, the specific capacity of P‐LLO and M‐LLO samples is 267.9 and 269.7 mAh/g, respectively. Furthermore, the cycling performance of the two samples is assessed at a current of 1 C, shown in **Figure** **2**a,d. The P‐LLO cathode shows poor capacity retention of 75.69% and severe average voltage decay of 332 mV after 100 cycles, indicating the occurrence of irreversible TM migration and structural degradation.^[^ ^20^ ^]^ In comparison, the M‐LLO sample exhibits better capacity stability (95.10%) and lower voltage drop (224 mV), implying that the structural stability is effectively enhanced. Meanwhile, the charge‐discharge CV profiles of 100 cycles in Figure 2b,c also reveal more stable capacity retention and mitigated voltage decay in the M‐LLO cathode.

In addition, the shift of the redox reaction potential in LLO cathodes is another important indicator of the irreversible phase transition.^[^ ^9b^ ^]^ To further investigate the influence of Mo treatment on the redox behavior, the corresponding differential capacity vs. voltage (*dQ/dV* ) curves for P‐LLO and M‐LLO samples are plotted in Figure 2e,f. For both samples, the peak pair in the region ≈ 4.0 V region is attributed to the Ni^2+^/^4+^ redox, which gradually weakens with cycling, due to the deactivation of Ni ions to form the rocksalt NiO phase.^[^ ^21^ ^]^ The other peak couple at ≈ 3.0 V is ascribed to the redox of Mn^4+^/^3+^ in the layered structure and spinel phase.^[^ ^22^ ^]^ With further cycling, the reduction peaks of Mn^4+^/^3+^ gradually shift to a lower potential, implying an increased spinel content. For the P‐LLO sample, the reduction potential of Mn^4+^/^3+^ shows a large shift toward 2.75 V, while the redox peak of Ni^2+^/^4+^ is significantly reduced. In contrast, the M‐LLO sample still maintains a strong redox peak of Ni^2+^/^4+^, and the reduction potential of Mn^4+^/^3+^ remains at ≈ 3 V after 100 cycles. The above improvement of the electrochemical performance confirms that the M‐LLO cathode shows a more stable crystal structure accompanied by the suppressed irreversible phase transition.^[^ ^23^ ^]^

### 2.3 Surface Structural and Electronic Evolution

**Figure 3:**
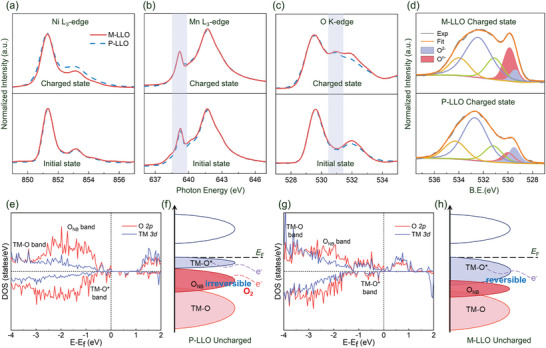
The a) Ni L‐edge, b) Mn L‐edge, and c) O K‐edge sXAS curves in TEY mode of the P‐LLO and M‐LLO samples at initial and charged states. The purple region in O K‐edge curves represents the non‐bonding O 2p orbitals located at ≈ 530.8 eV. d) O 1s XPS spectra of the P‐LLO and M‐LLO samples at charged states. e) The pDOS curves and f) the corresponding schematic diagrams of the P‐LLO surface models. g) The pDOS curves and h) the corresponding schematic diagrams of the M‐LLO surface models.

To explore the surface electronic structure changes, soft X‐ray absorption spectroscopy (sXAS) spectra in total electron yield (TEY) mode are employed based on P‐LLO and M‐LLO samples at initial and charged states. The Ni, Mn *L*‐edge s‐XAS spectra are normalized and shown in **Figure** **3**a,b, where it is widely recognized that an increase in the relative intensity of the peak located at lower binding energies indicates a decrease in the valence state of the corresponding metal.^[^ ^10^, ^24^ ^]^ In the initial state (bottom subgraphs), the valence state of Ni in both samples is the same, while the valence state of Mn is slightly decreased in the M‐LLO sample. The reason behind this is that when surface Li^+^ is occupied by high‐valence Ni, the valence state of the TMs will decrease to counterbalance the charge. As Mn has a lower redox potential of Mn^4+^/Mn^3+^ in comparison to Ni, the valence state of Mn will be comparatively lower.^[^ ^10^, ^14^ ^]^ In the charged state (top subgraphs), the valence states of Mn of the two samples remain the same, while the valence state of Ni in the M‐LLO is relatively low. This is because the decrease in the Li_2_MnO_3_ phase on the surface of M‐LLO leads to a lower capacity, wherein a portion of the capacity is taken up by Mn oxidation, thereby reducing the degree of Ni oxidation. The oxygen electronic structure evolution of the P‐LLO and M‐LLO samples at initial and charged states is also confirmed by the O *K*‐edge sXAS spectra. As shown in Figure 3c, the peaks at 529 eV and 532 eV are found in both samples, which is related to the number of electron holes of TM (Ni, Mn) 3d‐O 2p hybrid orbitals, that are, the electron holes on *t*_2g_ (529 eV), up‐spin electron holes on *e*_g_ (529 eV), and up‐down electrons holes on *e*_g_ (532 eV).^[^ ^14^, ^25^ ^]^ Besides, M‐LLO samples exhibit relatively higher peak intensity at 532 eV in both the initial and charged states. Based on the orbital analysis results in Supplementary Note, the higher peak intensity at 532 eV in M‐LLO is related to the surface Ni‐enrichment, which increases the ratio of electron holes (532 eV: 529 eV). Moreover, a new shoulder peak appears at 530.8 eV in the charged state in both samples, which is attributed to empty non‐bonding O 2p orbitals, implying the occurrence of OR reaction.^[^ ^26^ ^]^ In addition, X‐ray photoelectron spectroscopy (XPS) is also performed on both samples at initial and charged states to probe the oxygen electronic state at the cathode surface. As shown in Figure S5 (Supporting Information) and Figure 3d, the peaks located at ≈ 529.5 eV can be assigned to the lattice O^2−^, whereas the peaks located at higher binding energies (> 531 eV) are ascribed to the surface oxygen adsorbates.^[^ ^27^ ^]^ When charging to 4.7 V, both the P‐LLO and M‐LLO cathodes exhibit an additional peak centered at ≈ 530 eV, which is recognized as the lattice oxidized O_2_ *^n^* ^−^, implying the lattice oxygen on the surface is oxidized.^[^ ^28^ ^]^ Notably, there is a higher content of the O_2_ *^n^* ^–^ on the surface of the P‐LLO compared to M‐LLO, indicating that the OR process on the surface of P‐LLO occurs more vigorously. The drastic OR process on the surface is not conducive to the reversible oxygen reaction, which easily leads to the lattice oxygen loss and O_2_ release of the cathodes from the outside to the inside.^[^ ^29^ ^]^ In contrast, in M‐LLO, a considerable portion of the oxygen is still in the O^2−^ state and the state of surface oxygen atoms is more stable, which is attributed to the passivation effect of Nienrichment on surface oxygen.

Furthermore, Density functional theory (DFT) calculations are used to establish the intrinsic link between the OR reaction and the surface structure during the de‐lithiated process. First, for P‐LLO surface models (Li_x_Ni_0.2_Mn_0.6_O_2_, where x ranges from 1.2 to 0.1) in Figure S6 (Supporting Information), there is no O─O dimers observed on the surface when *x* > 0.6. Thereafter, severe O─O contraction and even O_2_ are found on the crystal surface when *x* ≤ 0.6. The reason is that the O atoms on the surface are less coordinated with TM atoms, so the TM─O bond cannot support the crystal structure at high voltage, resulting in the de‐coordination of the O atoms from the surface. Notably, the severe O─O contraction cannot be suppressed by Mo doping in Figure S7 (Supporting Information), because the unsaturated coordinated O atoms are widely present on the surface. For the M‐LLO surface models (Li_x_Ni_0.9_Mn_0.6_O_2_, where x ranges from 0.5 to 0.1) in Figure S8 (Supporting Information), no severe O─O contraction or oxygen gas is observed during the entire charging process in the M‐LLO models. This is because some of the Li on the surface is replaced by the Ni element. Hence, the oxygen atoms are trapped by surrounding metal atoms at high charging voltage and cannot be detached from the surface. In addition, the partial density of states (pDOS) and Bader charge in Figure 3e,g and Figure S9 (Supporting Information) are used to elucidate the electronic origin of the P‐LLO and M‐LLO models. For the initial P‐LLO model, the TM─O* antibonding band is narrow and the non‐bonding O 2p (O_NB_ in the pDOS figure) band is very close to the Fermi level. In contrast, the O_NB_ band of the M‐LLO model shifted to deeper energy levels because the extra Ni atoms coordinate with non‐bonding O atoms. Therefore, as shown in the schematic diagram Figure 3f, electrons can be very easily extracted from the O_NB_ band in the P‐LLO sample surface when a small amount of Li is extracted, causing severe O─O contractions and even O_2_ are generated directly on the surface. For the M‐LLO sample in Figure 3h, it is difficult to directly extract electrons from the downshifted O_NB_ band, so the surface O is protected. Furthermore, the Bader charge results in Figure S9 (Supporting Information) indicate that the number of lost electrons of Mn in M‐LLO in the initial state is lower than that of P‐LLO. In contrast, the average net Bader charge of the Ni element in both models remains almost identical, indicating that the Mn valence in M‐LLO is reduced while the Ni valence remains constant. Upon charging, the average number of lost Ni electrons in M‐LLO is notably lower than in P‐LLO, whereas the number of lost Mn electrons in both models is relatively similar. It suggests that Ni in M‐LLO is only partially oxidized compared to that in the P‐LLO model and that the final oxidation state of Mn is consistent in both models. Consequently, our Bader charge results are consistent with the sXAS findings. Briefly, the Ni‐enrichment structure makes the surface oxygen atoms fully coordinated with TM atoms, leading to a partial passivation of the OR reaction. Benefiting from this, the surface lattice oxygen framework under high voltages can be maintained, which inhibits the irreversible O_2_ release. Meanwhile, the redox process of cations through DFT calculations is confirmed to be consistent with the experimental process.

### 2.4 Interior Structural and Electronic Evolution

**Figure 4:**
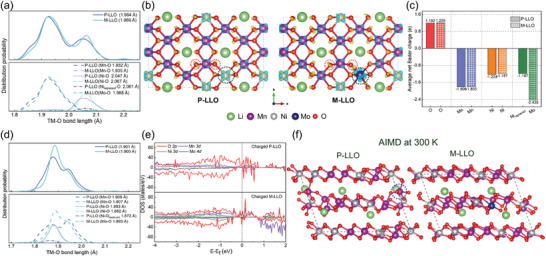
a) The distribution of the TM─O bond length for both lithiated P‐LLO and M‐LLO models, where the calculated average bond lengths are shown in the legends. b) The valence electron differential charge density for both lithiated P‐LLO and M‐LLO models, where the selected O atoms, replaced Ni atom and Mo atom are highlighted by red and black dotted lines, respectively; the blue region shows the electron loss and the yellow region shows the electron accumulation; the value of the iso‐surface is set to be 0.040 e Å^−3^ c) The average net Bader charge for both uncharged P‐LLO and M‐LLO models, where the minus and plus signs represent the electron loss and gain, respectively. d) The distribution of the TM─O bond length for both de‐lithiated P‐LLO and M‐LLO models, where the calculated average bond lengths are shown in the legends. e) The pDOS of de‐lithiated P‐LLO and M‐LLO models. f) The structure of de‐lithiated P‐LLO and M‐LLO models after the AIMD process at 300 K, 15 ps, and NVT ensemble, where the blue dotted circle is used to highlight the migration of the Mn atom.

The electronic properties of the interior structure are also investigated. First, the bulk P‐LLO and M‐LLO models are built and optimized by DFT calculations, where the optimal substituted site (Ni site) of the Mo atom in the M‐LLO model is determined by the formation energy (Figure S10, Supporting Information). Then the distribution curves and average values of Ni─O, Mn─O, Mo─O, and their sum TM─O bond lengths for the bulk P‐LLO and M‐LLO models in the initial state are plotted and listed in **Figure** **4**a. The results show that the average value of the TM─O bond length in the M‐LLO model (1.969 Å) is larger than that in the P‐LLO model (1.964 Å) in the initial states, which is mainly due to the longer Ni─O bonds in the M‐LLO model. Next, to explain this phenomenon, the valence electron differential charge density (Figure 4b) and Bader charge (Figure 4c) are calculated. As shown in Figure 4b, the charge‐depletion regions (blue) appear around the TM atoms, and the charge‐accumulation regions (yellow) are concentrated around the O atoms. Notably, there are more electrons accumulation of O atoms (red dotted circle) in the M‐LLO model compared to that of P‐LLO, which is attributed to more electrons depletion of the Mo atom (black dotted circle) in M‐LLO than that of the replaced Ni (Ni_replaced_) atom in P‐LLO models. In Figure 4c, the Bader charge is used to quantify the charge transfer numbers for all atoms. The results show that Mo atoms can transfer more electrons than Ni and Mn, and correspondingly, more electrons are localized around O atoms in the M‐LLO model. In addition, the average number of charge transfers of Mn atoms is almost the same in both models, meaning that they have similar valence states and ionic radii, resulting in similar bond length distributions. Besides, the average number of electron transfers of the Ni atoms is lower in the M‐LLO model, which indicates the valence of Ni is lower, resulting in a longer bond length.

Next, the optimized structural models of the P‐LLO and M‐LLO during the de‐lithiation process (Li_x_Ni_0.2_Mn_0.6_O_2_, where x ranges from 1.2 to 0.1) are shown in Figures S11 and S12 (Supporting Information). It is found that the intact crystal structures of both models are preserved. Moreover, the Bader charge results in Figures S13 and S14 (Supporting Information) demonstrate that TM atoms continuously lose electrons when x > 0.3. Meanwhile, the average number of electrons transferred by Ni atoms is higher in M‐LLO models, while that of Mn atoms is consistent for both models. When x ≤ 0.3, no electrons are transferred from TM atoms, which means that electrons are only lost from O atoms at high voltages. To reveal the veil of the OR reaction and further explore the relationship between bond length and electronic structure at high voltages, the de‐lithiation models with x = 0.1 are selected.

In Figure 4d, the bond length of TM─O becomes smaller after de‐lithiation, and the average bond length of M‐LLO is slightly lower than that of P‐LLO, which is consistent with the results of PDF in the charged states. Based on the structural model, the corresponding band properties, i.e., pDOS are further calculated and displayed in Figure 4e. The results show that the O 2p band is hybridized with the TM *d* band in M‐LLO near the Fermi level, implying stronger covalent properties. This results in stronger TM─O bonds, which will stabilize the O_2_^n−^ species during the OR reaction. On the contrary, the p‐DOS of P‐LLO exhibits the isolated O 2p band mainly located near the Fermi level, implying weak TM─O bonds that cannot stabilize the O_2_^n−^ species during the OR reaction. In particular, the neutron PDF results in Figure S15 (Supporting Information) also confirm that the short O─O pair distance in the M‐LLO sample is increased compared to that of P‐LLO at high voltages, implying strong TM─O bonds in the M‐LLO sample with moderate O─O distance contraction. To explore how Mo affects band hybridization, corresponding electronic properties for both models are calculated. The Bader charge and valence electron differential charge density results in Figure S16 (Supporting Information), demonstrate that the O atoms on the M‐LLO still retain more charge, which is beneficial to compensate for the electron extraction of O atoms. Moreover, the electron localized function (ELF) is used to analyze the localization status of electrons. ELF result in Figure S17 (Supporting Information) shows that more delocalized electrons are found around Mo, Mn, and O atoms in the M‐LLO model compared to that in the P‐LLO model, indicating that more electrons overlap between TM and O atoms leading to stronger TM─O covalent bonds in the M‐LLO model. According to the previous report, highly localized electrons of TM inhibit the circulation of electrons globally, making the electrons of the nearby atoms localized.^[^ ^30^ ^]^ On the contrary, the delocalized electrons around TM atoms can promote the interconnection of nearby TM and O valence electron domains. Here, in Figure S17 (Supporting Information), more electron transfer increases the number of delocalized electrons between Mo and O, promoting the Mn─O hybridizations in the charged states, leading to short and enhanced covalent bonds. Hence, the doping Mo atom can regulate the band hybridization of the O 2p band and TM *d* band, which can stabilize the TM‐(O_2_)^n−^ structure at high voltages. To more intuitively demonstrate the effect of stable TM‐(O_2_)^n−^ structure on TM migration, we simulated the migration process of TM ions at 300 K and 600 K by the ab initio molecular dynamic (AIMD) method, noting that high temperature is used to accelerate the migration process.^[^ ^31^ ^]^ The results in Figure 4f show that Mn migration is directly observed in the P‐LLO model at 300 K (blue circle). Furthermore, the migration of Mn is more obvious at 600 K in the P‐LLO model (Figure S18, Supporting Information). In contrast, M‐LLO shows a stable TM layer structure without the Mn migration at both 300 K and 600 K.

In short, the Mo doping leads to longer TM─O bonds in the uncharged model and shorter TM─O bonds in the charged model, in accordance with the PDF findings. Additionally, DFT calculations validate that more charge transfer between Mo and O produces delocalized electrons that enhance the electron delocalization of surrounding Mn and O atoms, leading to enhanced electron hybridizations and the formation of shorter and stronger covalent bonds at high voltages. Consequently, Mo doping can manipulate the band hybridization of the TM *d*‐O 2p, which stabilizes the TM‐(O_2_)*^n^* ^–^ species and inhibits TM migration.

### 2.5 In Situ Structure Evolution and Gas Release

**Figure 5:**
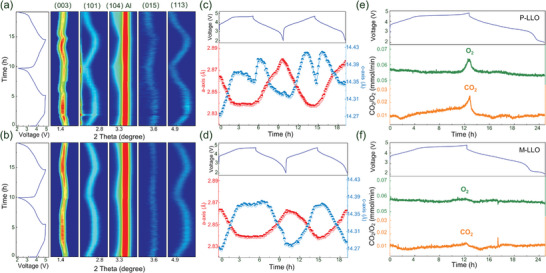
In situ characterizations. Contour plots of the synchrotron high‐energy XRD patterns along with the relevant voltage profiles for a) P‐LLO and b) M‐LLO samples. The evolution of lattice parameters extracted along the voltage profiles for c) P‐LLO and d) M‐LLO samples. Gas release of the a) P‐LLO and b) M‐LLO samples measured by in situ DEMS along with the corresponding voltage curves.

In situ XRD and DEMS are employed to characterize the evolution of the crystal structure and oxygen on both P‐LLO and M‐LLO samples during the charge‐discharge process.^[^ ^17^, ^32^ ^]^ The 2D contour plots in **Figure** **5**a,b depict the continuous lattice evolution of the characteristic peaks for P‐LLO and M‐LLO samples, corresponding to the voltage curves during the initial two cycles. Since the characteristic superlattice peaks of the *C2/m* phase are covered by the strong background signals during the in‐situ experiments, the obtained patterns can be fitted based on the *R‐3m* phase to facilitate the extraction of the lattice parameters (as shown in Figure 5c,d). During the initial charging process, both P‐LLO and M‐LLO exhibit contraction along the *a*‐axis, corresponding to the reduction in TM ion radius caused by cationic oxidation.^[^ ^33^ ^]^ The continuous expansion of the *c*‐axis at this stage is attributed to the extraction of lithium accompanied by the increased O─O interlayer repulsion.^[^ ^34^ ^]^ When the voltage further increases to 4.5 V, the cation reaction is basically completed with the unchanged lattice parameter, and the anion reaction dominates the charge compensation. At this stage, the value of *c* is affected by two mutually restraining factors: the gradual de‐lithiation process will increase the O─O repulsion to promote expansion, while the OR process will promote contraction. For the P‐LLO sample, there is an obvious contraction of the *c*‐axis followed by extreme expansion in high‐voltage regions, which is attributed to the destruction of the crystal structure by the violent irreversible oxygen release and TM migration. In contrast, the lattice parameter *c* of M‐LLO remains almost unchanged, indicating the contraction caused by OR process and the expansion of the de‐lithiation process have reached a balance, maintaining the stability of the crystal structure.^[^ ^34^ ^]^ During the discharging process, both two samples start to expand along the *a*‐axis and contract along the *c*‐axis, corresponding to the back‐insertion of lithium and the reduction reactions of TM and oxygen. Notably, the *a* and *c* lattice parameters of P‐LLO in the discharged state are significantly different compared to the initial state, indicating an irreversible distortion of the crystal structure, which may be attributed to the loss of lattice oxygen and irreversible TM migration.^[^ ^35^ ^]^ In contrast, the *a* and *c* parameters of M‐LLO almost recover to their initial values even after two cycles, demonstrating the reversibility of its crystal structure evolution. The in situ XRD results demonstrate the reversible OR process and inhabited TM migration in the M‐LLO cathode, benefiting from stable M‐(O_2_)*^n^* ^–^ species at high voltages by Mo doping. In situ DEMS intuitively detects the gas release during the first cycle of charge and discharge, which reflects the reversibility of the oxygen reaction and the stability of the surface structure. Figure 5e,f shows that M‐LLO has a significantly reduced detectable O_2_ and CO_2_ release in the high voltage regions compared to P‐LLO, indicating that irreversible oxygen loss and surface side reactions are significantly suppressed.^[^ ^36^ ^]^ Based on the in situ DEMS results, we confirm that the highly reactive surface O atoms are protected by the Ni‐enrichment structure, which hinders irreversible oxygen reactions penetration into the bulk phase.

## 3 Conclusion

In summary, we systematically investigate the effects of surface and interior electronic regulation on OR stabilization in LLO cathodes. On the surface of the M‐LLO sample, the oxygen is fully coordinated by TM ions due to the 3 nm Ni‐enrichment structure, causing the passivation of lone‐pair O 2p electrons and inhabited O_2_ release. For the internal structure, the more charge transfer of Mo increases the number of delocalized electrons around TM and O atoms, improving the degree of Mn─O hybridization at high voltages, which leads to the formation of shorter and stronger covalent bonds. Hence, the interior electronic regulation can manipulate the band hybridization of the TM d‐O 2p that stabilizes the TM‐(O_2_)*^n^* ^–^ species and inhibits TM migration. Benefitting from the special structural design, the modified cathode is confirmed to achieve reversible structure evolution and suppressed oxygen release through the in situ measurement. Accordingly, the capacity and voltage stability of the modified samples are significantly improved. In this work, an efficient method is designed to enhance the oxygen stability in the LLOs cathode through the surface‐to‐interior electronic structure modulation, which provides a new pathway to optimize the Li‐rich cathodes.

## Conflict of Interest

The authors declare no conflict of interest.

## Supporting information

Supporting Information

## Data Availability

The data that support the findings of this study are available from the corresponding author upon reasonable request.
