# Supplementary material for: Regulating the Electron Distribution of Metal‐Oxygen for Enhanced Oxygen Stability in Li‐rich Layered Cathodes
Source: Adv Sci (Weinh). 2024 Apr 22;11(24):2307397. doi: 10.1002/advs.202307397 (PMC11199972; doi:10.1002/advs.202307397)
Supplement: Supplementary file 1 — Supporting Information [file ADVS-11-2307397-s001.pdf]

ADVANCED SCIENCE
Open Access

Supporting Information

for *Adv. Sci.*, DOI 10.1002/advs.202307397

Regulating the Electron Distribution of Metal-Oxygen for Enhanced Oxygen Stability in Li-rich Layered Cathodes

Zijia Yin, Jun Zhao, Dong Luo, Yi-Ying Chin, Chien-Te Chen, Huaican Chen, Wen Yin, Yu Tang, Tingting Yang, Jincan Ren, Tianyi Li, Kamila M. Wiaderek, Qingyu Kong, Jun Fan, He Zhu*, Yang Ren and Qi Liu**

Supplementary Information

Regulating the electron distribution of metal-oxygen for enhanced oxygen stability in Li-rich layered cathodes

Z. Yin, Y. Ren, Q. Liu
Department of Physics
City University of Hong Kong
Hong Kong 999077, P. R. China
and Shenzhen Research Institute
City University of Hong Kong
Shenzhen 518057, Guangdong, China
E-mail: qiliu63@cityu.edu.hk

J. Zhao, J. Fan
Department of Materials Science and Engineering
City University of Hong Kong
Hong Kong 999077, P. R. China
E-mail: junfan@cityu.edu.hk

D. Luo, Y. Tang, T. Yang, J. Ren
Department of Physics
City University of Hong Kong
Hong Kong 999077, P. R. China

Y.-Y. Chin
Department of Physics
National Chung Cheng University
No.168, Sec. 1, University Rd., Minhsiung, Chiayi 621301, Taiwan

C.-T. Chen
National Synchrotron Radiation Research Center
101 Hsin-Ann Road, Hsinchu 30076, Taiwan

H. Chen, W. Yin
Institute of High Energy Physics
Chinese Academy of Sciences (CAS)
Beijing 100049, People's Republic of China

T. Li, K. M. Wiaderek
X-Ray Science Division
Argonne National Laboratory
Lemont, IL 60439, USA

Q. Kong
Société Civile Synchrotron SOLEIL
L'Orme des Merisiers
Saint-Aubin, BP 48, 91192, GIF-sur-Yvette, CEDEX, France

H. Zhu,
Herbert Gleiter Institute of Nanoscience
School of Materials Science and Engineering, Nanjing University of Science and Technology, Nanjing 210094, China.
and Shenzhen Research Institute
City University of Hong Kong
Shenzhen 518057, Guangdong, China
E-mail: hezhu@njust.edu.cn

† These authors contributed equally: Zijia Yin and Jun Zhao.

Experimental section

Material synthesis

Preparation of pristine material

The Li-rich $Li_{1.2}Ni_{0.2}Mn_{0.6}O_2$ cathode materials were synthesized through molten salt assisted calcination method. Firstly, transition metal carbonate precursor was synthesized through carbonate precipitation method with a molar ratio of Mn: Ni = 0.6:0.2. An aqueous solution containing the required stoichiometric amounts of $MnSO_4$ and $NiSO_4$ was pumped into a 500 mL beaker with continuous stirring. 2M Na_2CO_3 solution with a controlled amount of NH_4OH as a chelating agent was simultaneously fed into the reactor. The temperature and pH of the solution were maintained at 55 °C and 8.0, respectively, throughout the coprecipitation reaction. The obtained $Ni_{0.2}Mn_{0.6}(CO3)_{0.8}$ precursor precipitate was filtered, washed, and dried in vacuum at 80 °C. Then, the precursor (0.927g), molten salt NaCl (1.87g), KCl (3.5784g) and lithium carbonate (0.4702g) (excess 5% to compensate for lithium loss under high temperature conditions) were ground and mixed uniformly. Afterwards, the mixture was transferred to the muffle furnace and calcined at 800 °C for 12 hours, and naturally cooled to room temperature. Finally, the pristine materials were washed by water, filtered, and dried at 180°C for 12 hours to obtain the final Li-rich $Li_{1.2}Ni_{0.2}Mn_{0.6}O_2$ cathode (marked as P-LLO).

Preparation of Mo-treatment material

Firstly, the carbonate precursor was synthesized through the similar method as mentioned. Then, $(NH_4)_6Mo_7O_{24} \cdot 7H_2O$ (0.0530g), precursor (0.927g) and 25mL deionized water were added into the 50mL Teflon reaction vessel. After stirring 30 minutes, the Teflon reaction vessel was transferred to oven, kept at 150 °C for 12h. Afterwards, the modified precursor was calcined with molten salt (NaCl, KCl) and lithium carbonate under the same procedure as the pristine materials. Finally, the modified materials were washed by water, filtered, and dried at 180°C for 12 hours to obtain the final Li-rich $Li_{1.2}Ni_{0.2}Mn_{0.6}O_2$ cathode (marked as M-LLO).

Electrochemical measurements

The electrochemical performances of the samples were measured using a CR2030 type coin cell. The cathode materials were prepared as follows: 80 wt% of the synthesized powders, 10 wt% of carbon black, and 10 wt% of polyvinylidene fluoride (PVDF) were mixed using N-methyl pyrrolidinone (NMP) as a solvent. The resulting slurry was coated on Al foil by a doctor blade technique and vacuum dried at 100 ^oC overnight. The cells were assembled in an Ar-filled glove box with the samples, lithium foil, a polymer separator and 1 M $LiPF_6$ in ethylene carbonate (EC): ethyl methyl carbonate (EMC): dimethyl carbonate (DMC) (1: 1: 1 by volume) as the electrolyte. The charge-discharge cycling was performed in the potential window of 2.0-4.7 V (vs. Li/Li^+) at 28 ^oC with different current densities of 20 and 200 mA g^{-1}.

Sample characterization

The atomic ratio of Li, Ni, Mn, and Mo were analyzed with ICPOES using Agilent 720ES. The surface structure and elemental distribution were measured by the scanning transmission electron microscopy (FEI Titan Cubed Themis G2300 STEM) equipped with a double-aberration corrector. High angle annular dark field (HAADF)-STEM imaging at atomic resolution (JEM-ARM200F) and energy dispersive X-ray spectroscopy (EDS) mapping were performed at 300 kV. In order to illustrate the changes of the lattice structure and local ligand geometry, powder XRD and pair distribution function (PDF) based on synchrotron total scattering data collection were conducted at the 11-ID-C beamline (λ =0.1173Å) of the Advanced Photon Source (APS), Argonne National Laboratory (ANL), U.S. The collected XRD patterns were refined based on the Rietveld method using Fullprof software. Besides, the scattering 2D patterns of the PDF were calibrated and integrated into 1D profiles by Fit 2D software, and then compute G(r) patterns through the Fourier transform. The refinement of the PDF profiles was conducted using PDFgui software based on the relevant structural model.[1] The soft X-ray absorption spectroscopy (SXAS) data of the Ni, Mn

L-edge and O *K*-edge were collected in both total electron yield (TEY) modes at the beamline (BL) 11 A of the National Synchrotron Radiation Research Center (NSRRC), Taiwan. MnO, $LaMnO_3$ and NiO were measured simultaneously to calibrate the energy scale. O 1 s spectra of the samples at initial and charged states were acquired on X-ray photoelectron spectroscopy (Thermo Scientific K-Alpha Nexsa). The binding energy values of the obtained spectra were calibrated using the C 1s peak at 284.8 eV. Neutron PDF experiments were performed on Multi-Physics Instrument (MPI) at China Spallation Neutron Source (CSNS), Dongguan, China. About 1g powder samples (pristine sample, carbon background and charged samples with carbon) were loaded into 9-mm quartz capillaries. The charged sample mixture was washed by dimethyl carbonate (DMC) to remove the binder and dried in a glovebox. After subtracting the signal of carbon based on the mass ratio, the obtained total scattering structure factor S(Q) data was further transformed into PDF G(r) data.

In situ synchrotron XRD characterizations were carried out using the 11-ID-C beamline (λ =0.1173Å) at the APS of Argonne National Laboratory. The adopted 2032 cell body was designed with two 3 mm holes on both sides of the cell and sealed with Kapton film for X-ray transmission. *In situ* cells were cycled at a constant current of 0.2C between 2.0-4.7 V *vs*. Li^+/Li. The collected XRD patterns were calibrated and integrated by Fit 2D software, and then the refined based on the Lebail method through Fullprof software.[2] *In situ* Differential Electrochemical Mass Spectrometer (DEMS) measurement was carried out to detect the gas evolution during first cycle. The DEMS cell was assembled with a commercial Swagelok-type cell in an Ar-filled glovebox, where the diameter and loading density of electrode disc were 14 mm and 5 mg cm^{-2}, respectively. Ar carrier gas flowed through the measuring cell at a rate of 3.6 ml min^{-1} and then connected with the mass spectrometer (QAS 100). After ventilating for 6 hours until a stable baseline, cells were cycled between 2.0-4.7 V at a specific current of 40 mA/g on a Neware battery test system (CT-4008T-5V 50 mA-164).

Density functional theory (DFT) methods

All DFT calculations of P-LLO and M-LLO models were conducted on Vienna ab initio simulation package (VASP) with the Projector-Augmented-Wave (PAW) method and Perdew-Burke-Ernzerhof (PBE) of the Generalized Gradient Approximation (GGA) exchange-correlation functional.[3] The cut-off energy for all models was set to 520 eV and the convergence criteria were set to 1×10^{-5} eV/cell of energy and 0.02 eV/Å of force, respectively. Spin-polarized calculation were considered in all tasks. To correct exchange correlation energy of TM atoms, the Hubbard U correction (GGA+U) method was applied where DFT+U values of Ni, Mn and Mo atoms were set to 6.2 eV, 3.9 eV, and 4.38 eV, respectively.[4] To simulate the TM migration process of the entire models, the *ab initio* molecular dynamics (AIMD) based on NVT ensemble at 300 K and 600 K were calculated, which can provide driving force for overcome migration energy barrier. The bond strength can be analyzed by the Bader charge[5] and crystal orbital hamiltonian population (COHP).[6]

For the surface model, according to the STEM result in **Figure 1**, models with 30 Å thickness were constructed to simulate experimental results with gradient properties. Besides, the atomic composition of the P-LLO model and the M-LLO model was $Li_{44}Ni_7Mn_{21}O_{72}$ and $Li_{22}Ni_{29}Mn_{21}O_{72}$, respectively, which was based on the ICP **(Table S1)** and EDX **(Figure S2)**. Meanwhile, 20 configurations including Li/Ni exchange and Ni/Mn exchange of the P-LLO model were considered, and the configuration with the lowest energy was selected. Besides, another Mo-doped surface model ($Li_{44}Ni_6Mn_{21}Mo_1O_{72}$) was constructed to explore the effect of Mo atoms on the surface. For the bulk models, the atomic composition of the P-LLO model and the M-LLO model was $Li_{43}Ni_8Mn_{21}O_{72}$ and $Li_{43}Ni_7Mn_{21}Mo_1O_{72}$, respectively, which was based on the ICP **(Table S1)** results. Meanwhile, 20 configurations including Li/Ni exchange and Ni/Mn exchange of the P-LLO model were considered, and the configuration with the lowest energy was selected. To find the optimal Mo-doped site, the formation energies were calculated based on all possible substituted sites in the bulk Mo-LLO model. The

formation energies (ΔE_{form}) for different Mo-doped sites are described by the following equations.

Mo dopant at Mn site:

$$\Delta E_{form} = E^{(Mo-doped)} - E^{(LLO)} - E^{(MoO_3)} + E^{(MnO_2)} - 1/2E^{(O_2)}$$

Mo dopant at Ni site:

$$\Delta E_{form} = E^{(Mo-doped)} - E^{(LLO)} - E^{(MoO_3)} + E^{(NiO)} + E^{(O_2)}$$

Mo dopant at Li1 (Li in TM layer) and Li2 (Li in Li layer) sites:

$$\Delta E_{form} = E^{(Mo-doped)} - E^{(LLO)} - E^{(MoO_3)} + 1/2E^{(Li_2O)} + 1.25E^{(O_2)}$$

where $E^{(Mo-doped)}$ is the total energy of the Mo-doped structure and $E^{(LLO)}$ is bulk pristine LLO structure, respectively; $E^{(MoO_3)}$, $E^{(MnO_2)}$, $E^{(NiO)}$ and $E^{(Li_2O)}$are the total energies of bulk MoO_3, MnO_2, NiO and Li_2O structures. To ensure that same amount of oxygen before and after reaction, $E^{(O_2)}$ is added to the equations.

Supplementary Figures and Tables

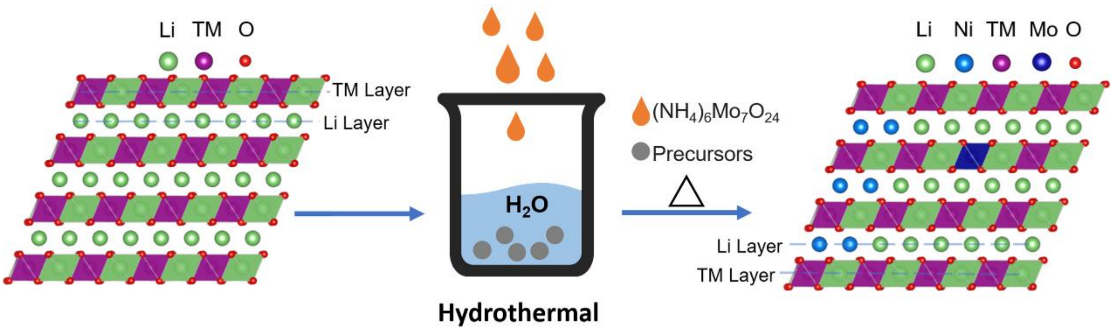

Figure S1. The schematic diagram of the preparation method of M-LLO sample.

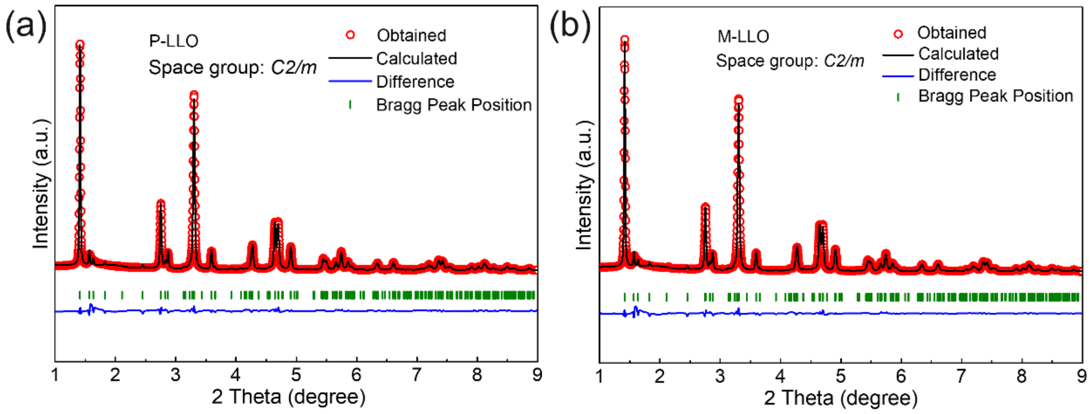

Figure S2. The XRD patterns and Rietveld refinement results of the (a) P-LLO and (b) M-LLO samples.

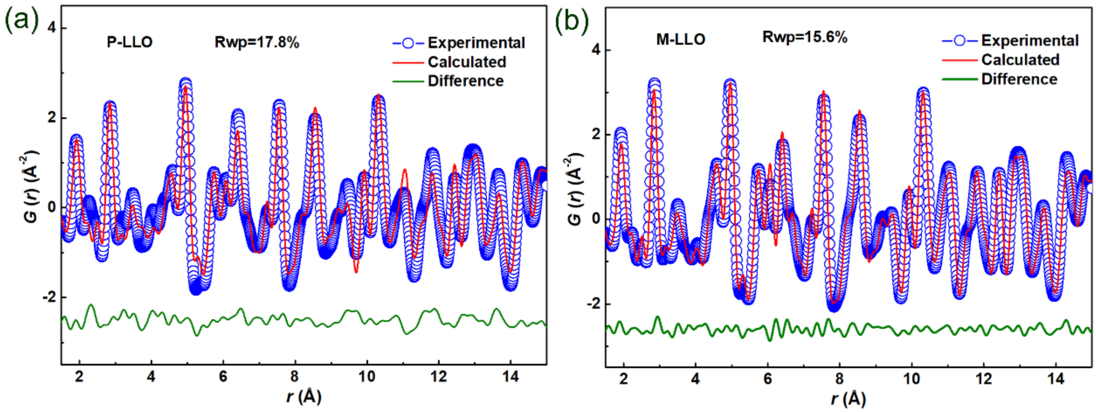

Figure S3. Pair distribution function (PDF) and refinement results patterns by the PDFgui analysis on (a) P-LLO and (b) M-LLO.

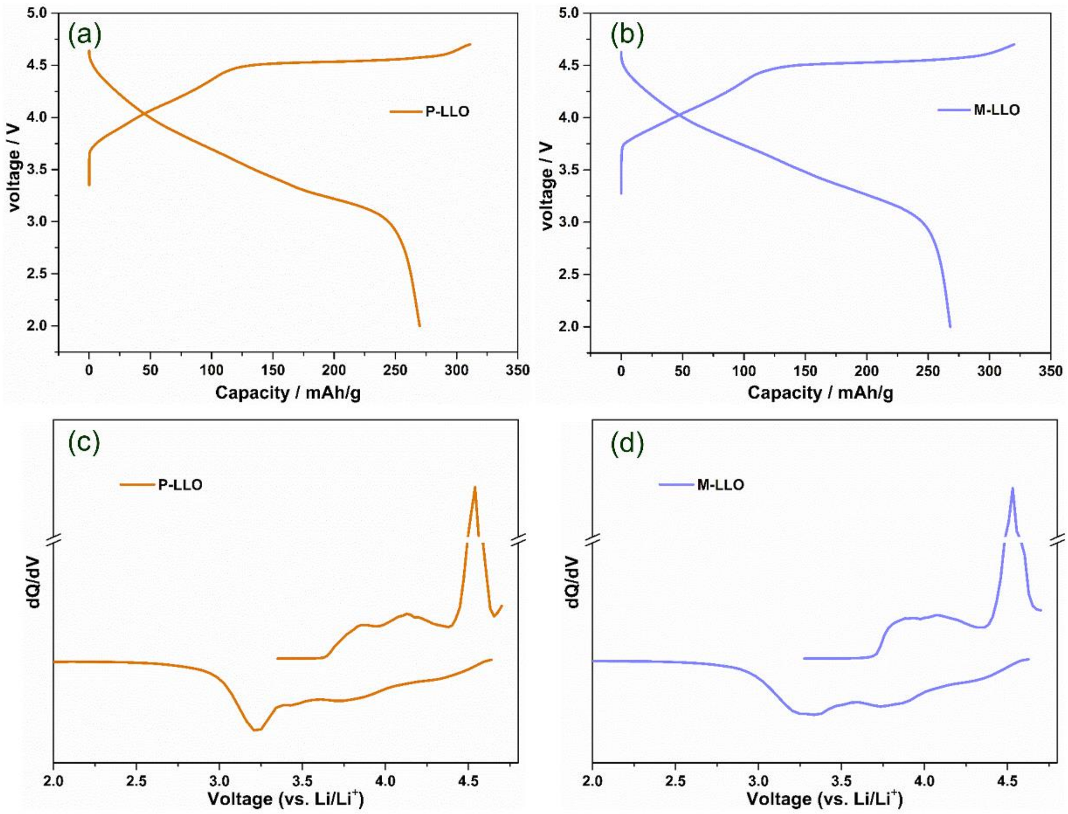

Figure S4. (a, b) Initial charge/discharge profiles at 0.1C rate for P-LLO and M-LLO samples between 2.0-4.7 V and (c, d) The corresponding dQ/dV curves of samples.

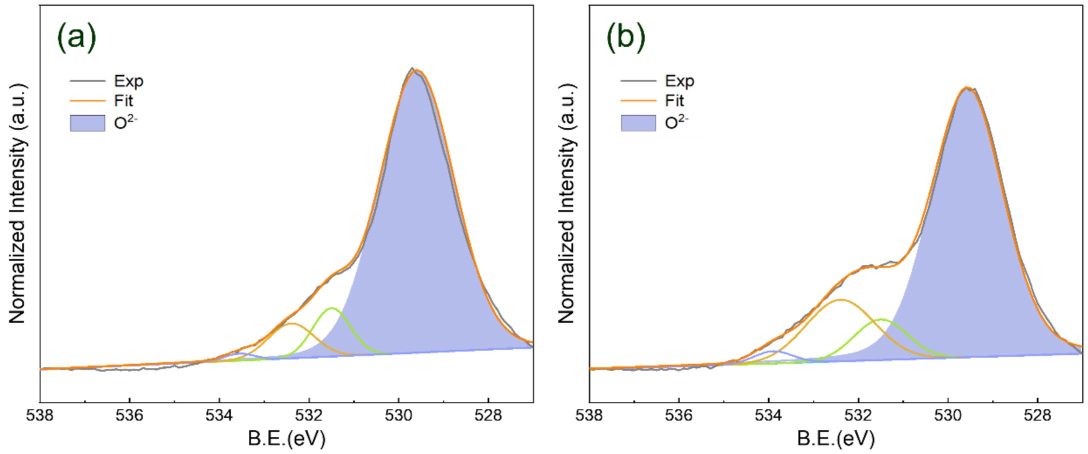

Figure S5. O 1s XPS spectra of the P-LLO and M-LLO sample at initial states.

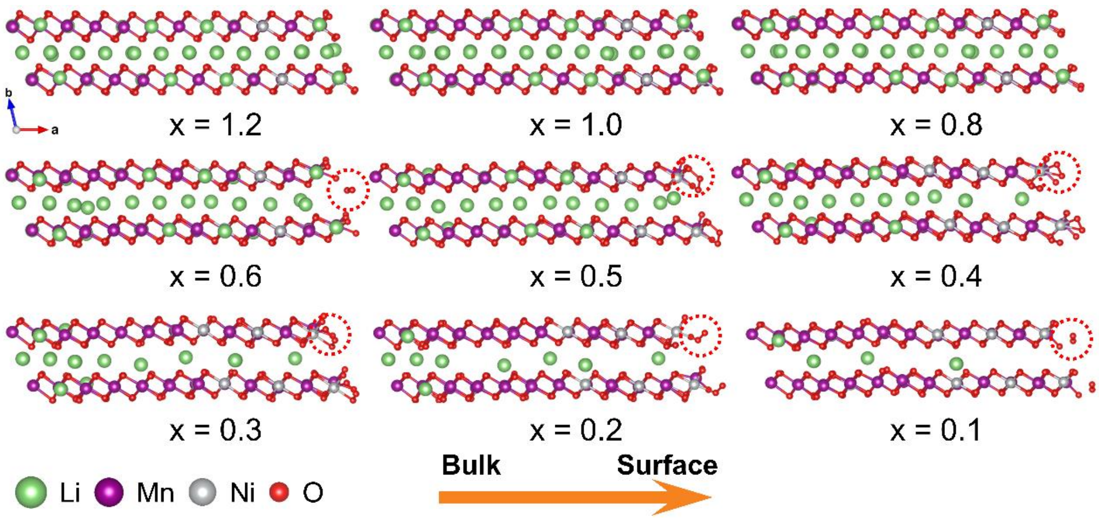

Figure S6. The figure shows the lithiated and de-lithiated P-LLO ($Li_xNi_{0.2}Mn_{0.6}O_2$, where x = 1.2, 1.0, 0.8, 0.6, 0.5, 0.4, 0.3, 0.2, and 0.1) surface models. The red dashed circles represent the O-O dimers or O_2 and orange arrows illustrate the model from left to right representing bulk to surface.

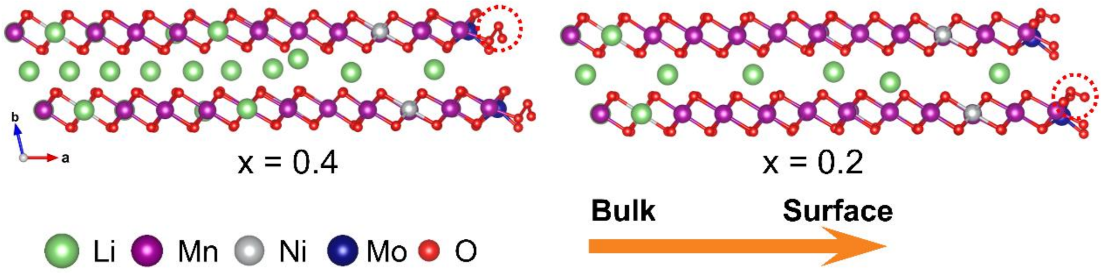

Figure S7. The lithiated and de-lithiated Mo-doped LLO (Mo-$Li_xNi_{0.2}Mn_{0.6}O_2$, where x = 0.4 and 0.2) surface models. The orange arrows illustrate the model from left to right representing bulk to surface.

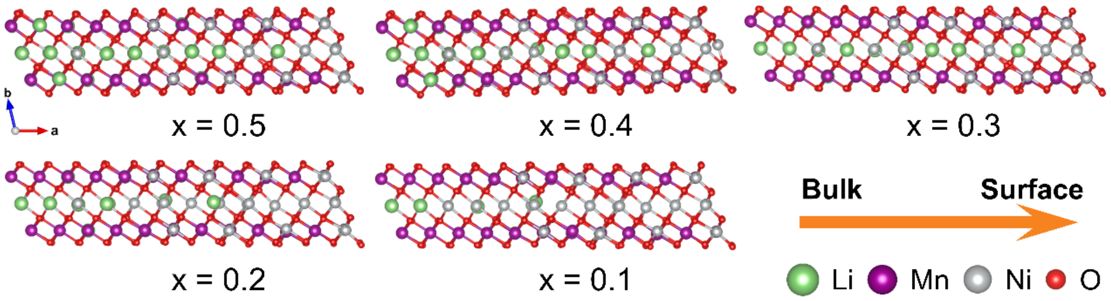

Figure S8. The figure shows the lithiated and de-lithiated M-LLO ($Li_xNi_{0.9}Mn_{0.6}O_2$, where x = 0.5, 0.4, 0.3, 0.2, and 0.1) surface models. The orange arrows illustrate the model from left to right representing bulk to surface.

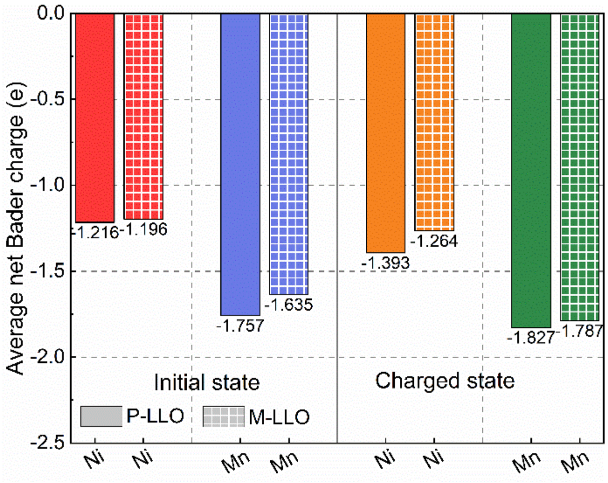

Figure S9. Net average Bader charge of Mn and Ni atoms in P-LLO and M-LLO models in the initial and charged state, where the minus sign represents the electron loss.

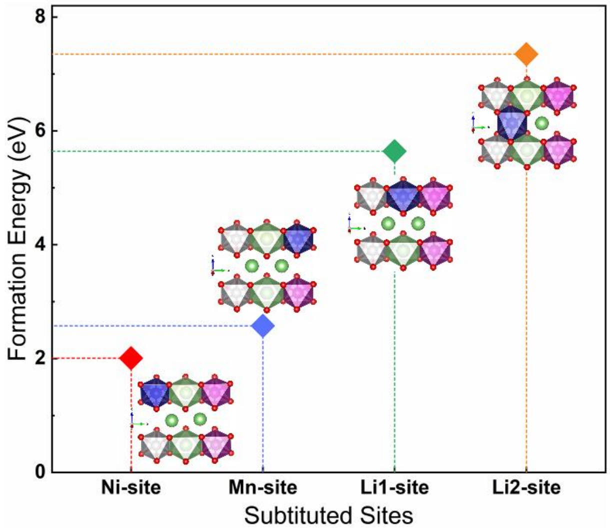

Figure S10. Formation energies of Mo-LLO models at different substituted sites. The inset shows the specific Mo-doped sites. The purple octahedrons, green spheres/octahedrons, grey octahedron, and blue octahedron represent the Mn octahedron, Li atoms /octahedrons, Ni octahedron, and Mo octahedron, respectively.

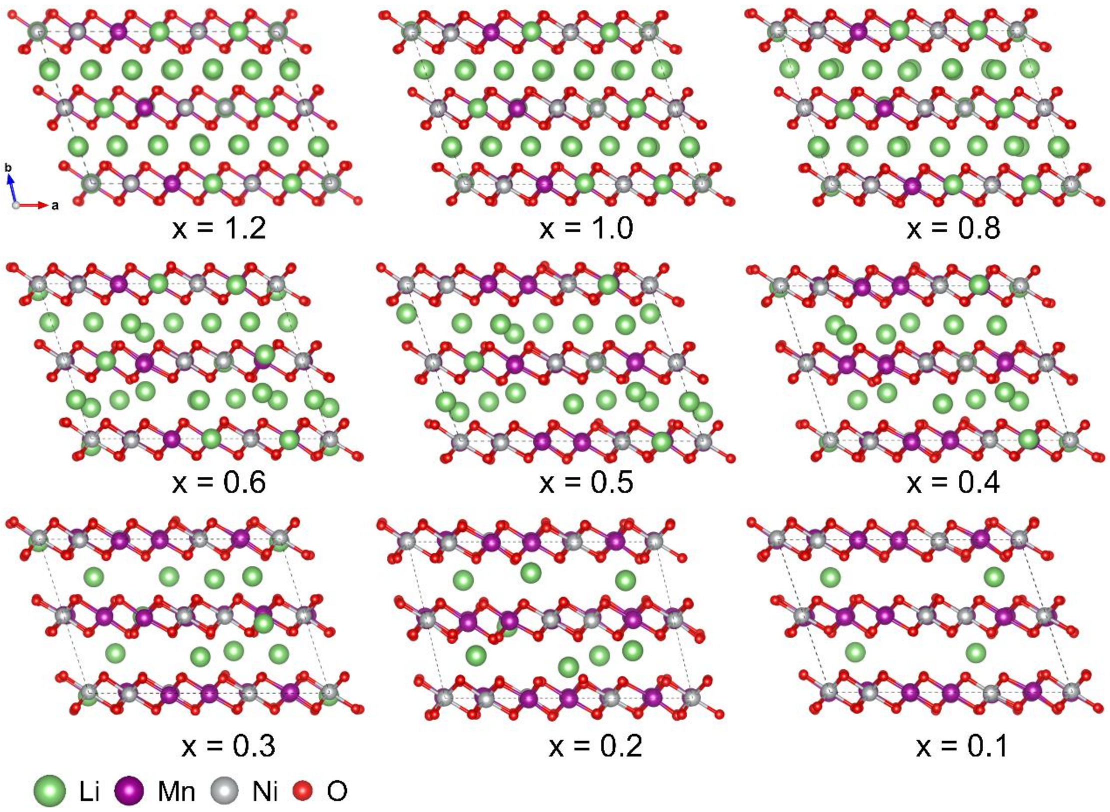

Figure S11. The de-lithiated bulk P-LLO ($Li_xNi_{0.2}Mn_{0.6}O_2$, where x = 1.2, 1.0, 0.8, 0.6, 0.5, 0.4, 0.3, 0.2, and 0.1) models.

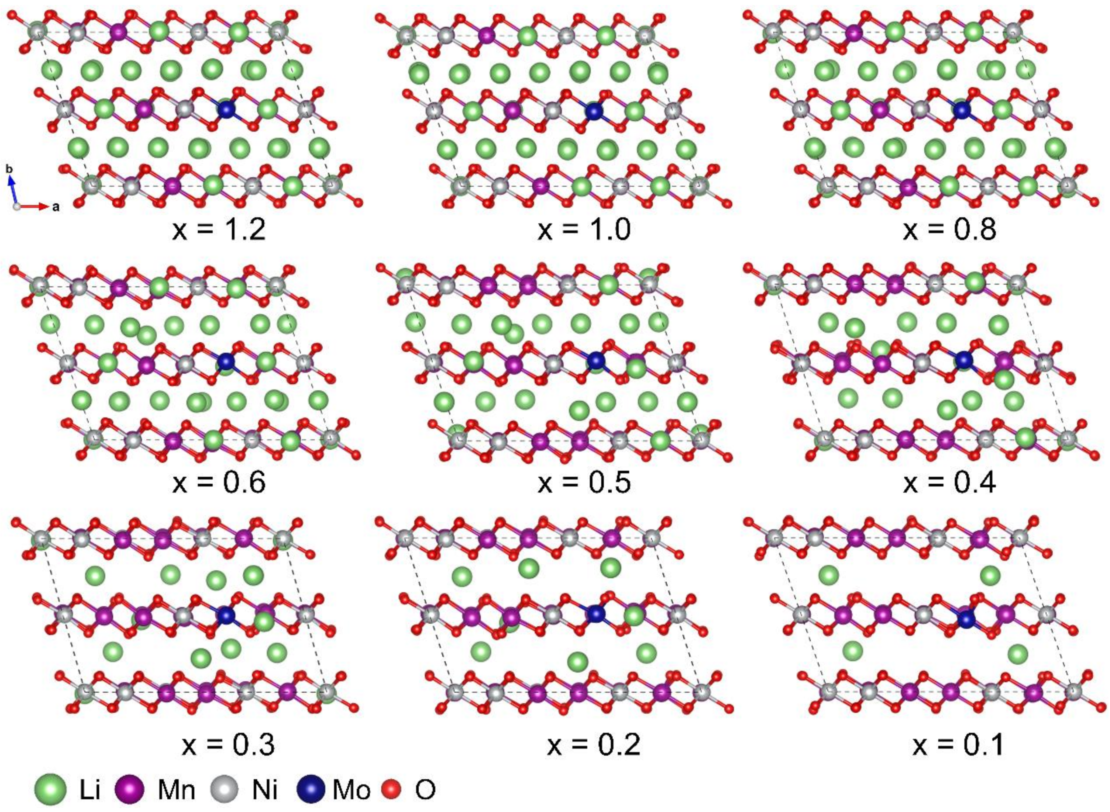

Figure S12. The de-lithiated bulk M-LLO (Mo-$Li_xNi_{0.2}Mn_{0.6}O_2$, where x = 1.2, 1.0, 0.8, 0.6, 0.5, 0.4, 0.3, 0.2, and 0.1) models.

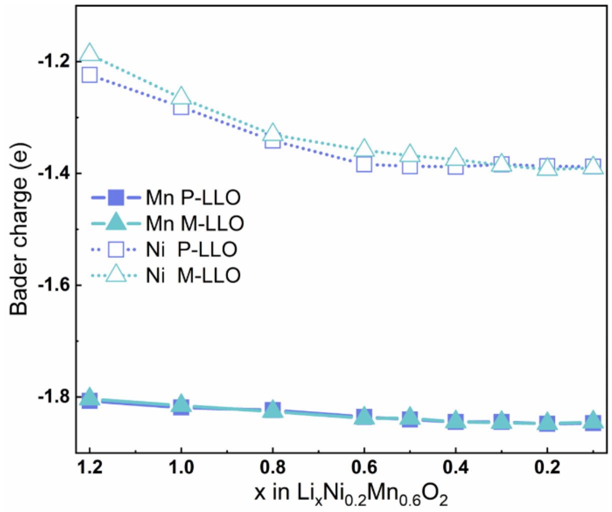

Figure S13. Net average Bader charge of Mn and Ni atoms in P-LLO and M-LLO models during the de-lithiation process, where the minus sign represents the electron loss.

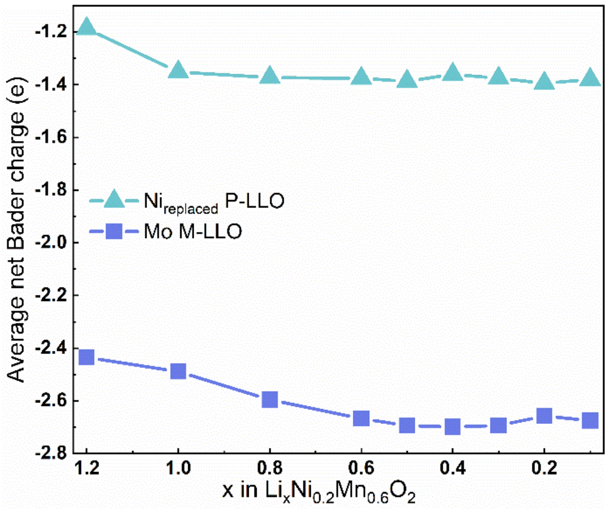

Figure S14. Net average Bader charge of $Ni_{replaced}$ and Mo atoms in P-LLO and M-LLO models during the de-lithiation process, where the minus sign represents the electron loss and $Ni_{replaced}$ represents this Ni site in P-LLO model is the same as Mo site in M-LLO model.

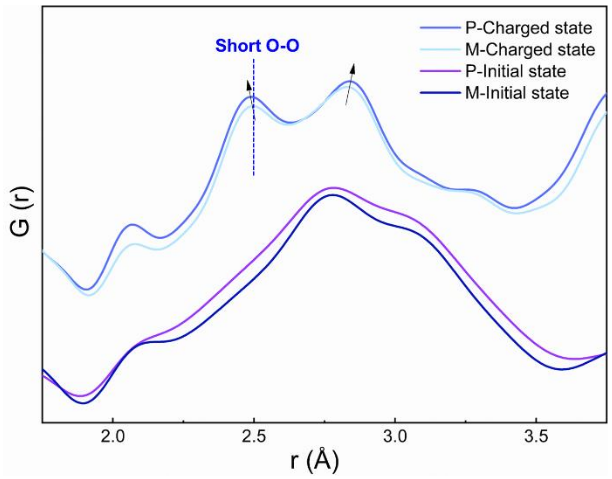

Figure S15. Comparison of the ex situ neutron PDF results of P-LLO and M-LLO collected at initial and charged states.

Note: According to previous report,[7] the O-O pair distance range from 2.5 Å to 3.2 Å of two samples at initial state is ascribe to the interlayer and intralayer O-O in TM-O octahedra, where the main peak is about 2.76 Å. For charged sample, the large amount of short O-O pairs appear at 2.3 Å-2.6 Å, which is due to the oxygen redox reaction that makes the interlayer O-O contraction. Moreover, the peak position of short O-O pair for the M-LLO sample is larger than that of the P-LLO sample, indicating that the short O-O pair distance in M-LLO sample is increased compared to that of P-LLO at high voltages.

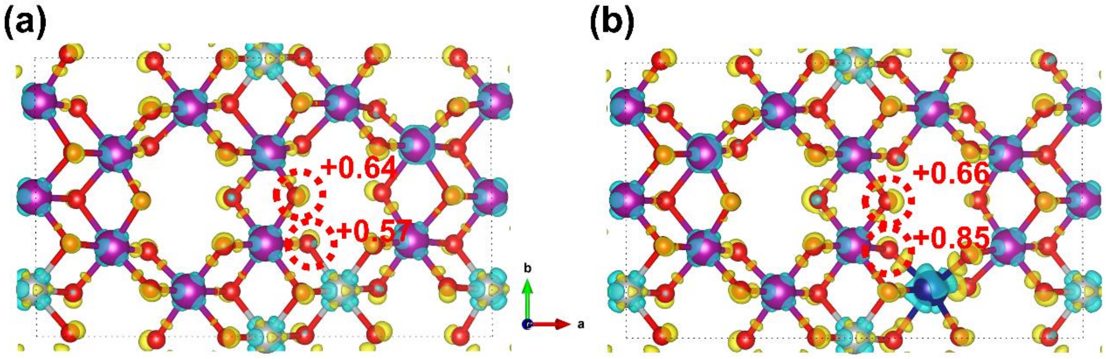

Figure S16. The valence electron differential charge density and Bader charge (red circles) for bulk P-LLO (a) and M-LLO (b) models, where the iso-surface is set to 0.035 e/Å^3 and the positive sign represents the obtained electrons.

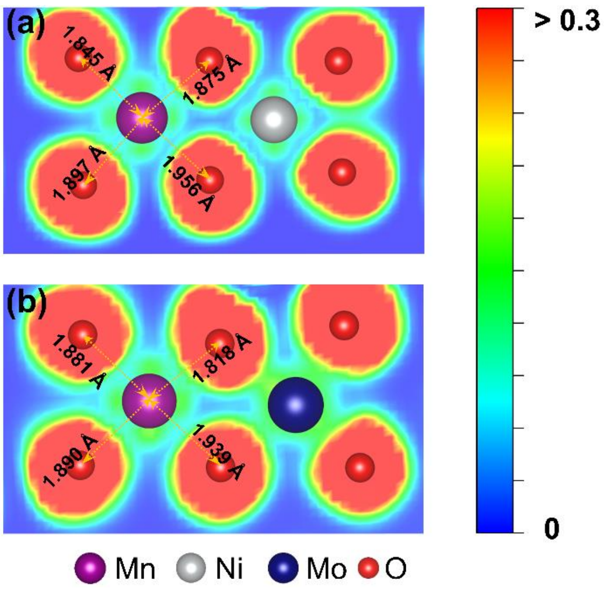

Figure S17. Electron localized function (ELF) diagram of the P-LLO model (a) and the M-LLO model (b), where ELF values of 0, and 1 represent completely delocalized electrons and completely localized electrons, respectively. In this ELF diagram, ELF = 0.3 is set to the maximum saturation for easy comparison.

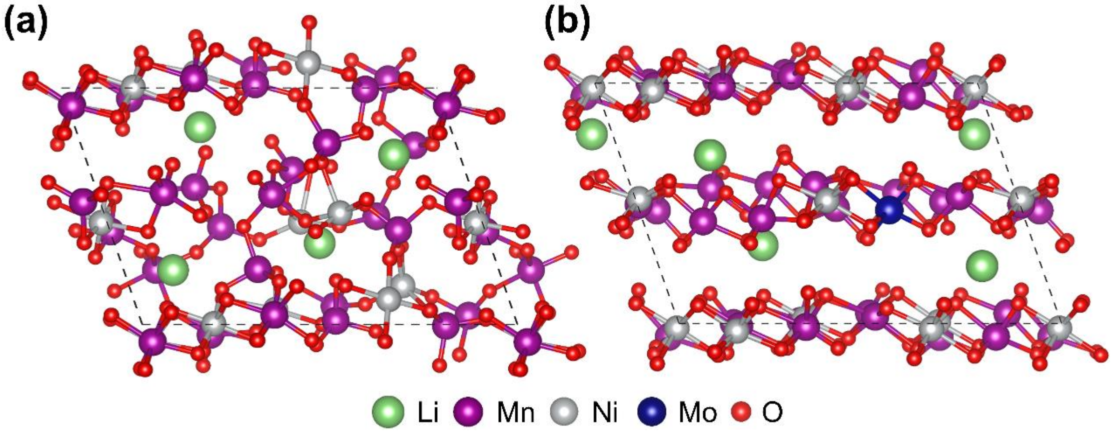

Figure S18. The structure of bulk P-LLO (a) and M-LLO (b) models after AIMD process at 600 K and NVT ensemble.

Table S1. The element distribution obtained from ICP results and STEM-EDS results.

Characterization	Samples	Element			
		Li	Ni	Mn	Mo
ICP	P-LLO	1.2036	0.2021	0.5952	0
	M-LLO	1.1987	0.2013	0.5988	0.0168
STEM-EDS	M-LLO	-	0.2*	0.5942	0.0172

* The data obtained by STEM-EDX is the atomic ratio of each element. We use Ni as 0.2 to convert the corresponding composition of Mn and Mo in the composition formula.

Table S2. Refined parameters for the P-LLO and M-LLO materials using ex situ synchrotron XRD (λ = 0.1173 Å). Monoclinic Li_2MnO_3 (space group: *C2/m*) was used as structural model.

Sample	Atom	Site	X	Y	Z	Occupancy
**P-LLO**	Mn1	4g	0	0.1694(9)	0	0.9
	Ni1	4g	0	0.1694(9)	0	0.0952(0)
a=4.9607(9) Å	Li1	4g	0	0.1694(9)	0	0.0048(0)
b=8.5768(8) Å	Ni2	2b	0	0.5	0	0.3979(0)
c=5.0368(8) Å	Li2	2b	0	0.5	0	0.6021(0)
β=109.3136(2)º	Li3	2c	0	0	0.5	0.9805(9)
	Ni3	2c	0	0	0.5	0.0194(1)
Rp=5.01%	Li4	4h	0	0.6911(3)	0.5	0.9609(3)
Rwp=7.86%	Ni4	4h	0	0.6910(7)	0.5	0.0390(7)
Rexp=1.49%	O1	4i	0.2093(2)	0	0.2187(9)	2
	O2	8j	0.2570(7)	0.3498(3)	0.2330(6)	2
**M-LLO**	Mn1	4g	0	0.1694(6)	0	0.9
	Ni1	4g	0	0.1694(6)	0	0.0946(6)
a=4.9628(8) Å	Li1	4g	0	0.1694(6)	0	0.0053(4)
b=8.5751(4) Å	Ni2	2b	0	0.5	0	0.3914(7)
c=5.0373(5) Å	Li2	2b	0	0.5	0	0.6085(3)
β=109.3734(9)º	Li3	2c	0	0	0.5	0.9789(5)
	Ni3	2c	0	0	0.5	0.0210(5)
Rp=4.80%	Li4	4h	0	0.6902(4)	0.5	0.9582(5)
Rwp=7.75%	Ni4	4h	0	0.6902(4)	0.5	0.0417(5)
Rexp=1.47%	O1	4i	0.2108(8)	0	0.2173(5)	2
	O2	8j	0.2579(8)	0.3495(4)	0.2332(0)	2

Supplementary Note

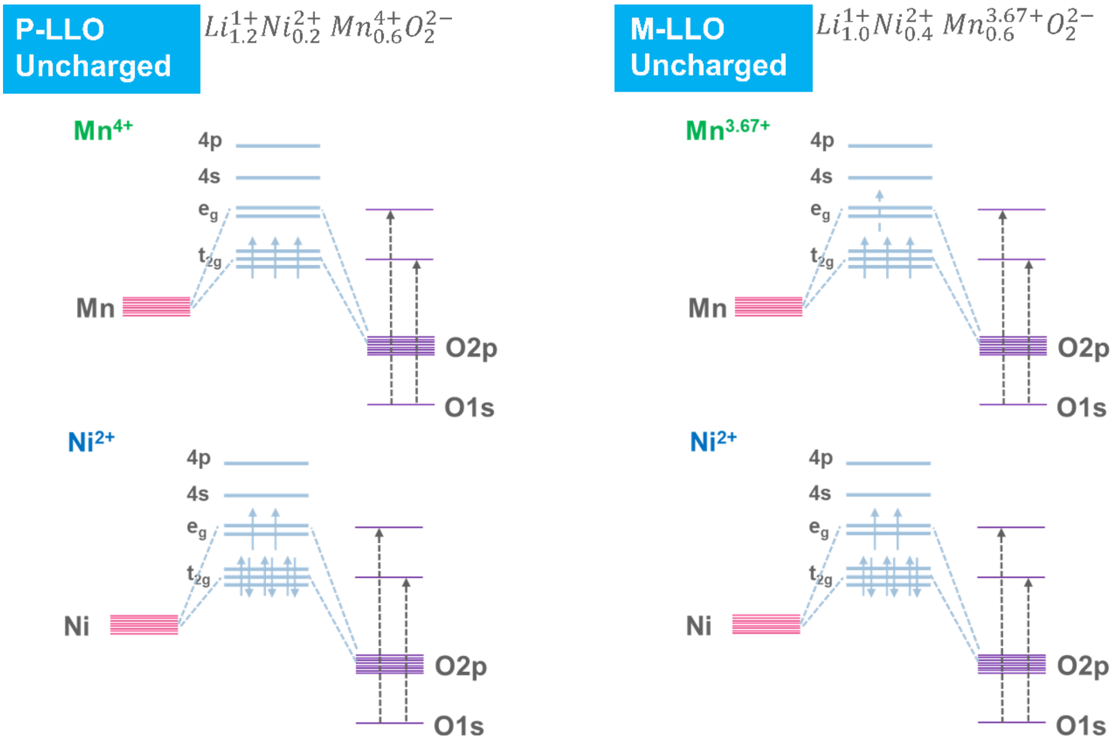

Figure Note1. The electronic arrangement of TM-O hybrid orbitals for uncharged P-LLO and M-LLO samples, where Mn is the high-spin system and Ni is the low-spin system.

According to previous report,[8] for the 3d TM system, the empty orbitals of t_{2g} and the spin up orbitals of e_g are classified as low-energy orbitals (529.7 eV) and the spin down orbitals of e_g are classified as high-energy orbitals (532 eV). Hence, the ratio of peak intensity at 529.7 eV and 532 eV can be obtained by calculating the number of the empty low- and high-energy orbitals based on the atomic ratio and valence of Mn and Ni elements.

For the uncharged P-LLO sample, the surface atomic ratio and valence can be easily determined by the chemical composition ($Li_{1.2}Ni_{0.2}Mn_{0.6}O_2$), as shown in the **Figure Note1** and **Table Note1**. Hence, there are five low-energy orbitals and two high-energy orbitals for the Mn^{4+} atom, and zero low-energy orbitals and two high-energy orbitals for the Ni^{2+} atom, causing the ratio of high-energy and low-energy orbitals is equal to 1:0.533. For the uncharged M-LLO sample, the atomic ratio for the special Ni-

segregation structure should be determined first. Because the detective limits of sXAS is about 10 nm depth, the atomic ratio of Ni and Mn in the whole 10 nm depth can be obtained from STEM-EDS results, and the corresponding surface chemical composition of M-LLO sample with a 10 nm thickness is $Li_{1.0}Ni_{0.4}Mn_{0.6}O_2$ shown in the **Figure Note1**. Next, the valence of Ni can be determined as 2+ since the Ni L-edge sXAS curve of the uncharged M-LLO sample is the same as that of the P-LLO sample. Hence, the valence of Mn can be calculated as 3.67+ according to the chemical composition of Ni-enrichment structure, which is in accordance with the Mn L-edge sXAS curve in **Figure 3a**. And then, there are 4.67 low-energy orbitals and two high-energy orbitals for the $Mn^{3.67+}$ atom, and two low-energy orbitals and two high-energy orbitals for the Ni^{2+} atom, causing the ratio of high-energy and low-energy orbitals is equal to 1:0.713. Finally, the peak intensity at 532 eV is higher in the uncharged M-LLO sample than that of the P-LLO sample.

For the charged P-LLO sample ($Li_{0.1}Ni_{0.2}Mn_{0.6}O_2$), the Ni atoms are oxidized to 4+ and Mn atoms are difficult to oxidized leading to unchanged valence, as shown in the **Figure Note2** and **Table Note1**. Therefore, the O atoms are oxidized to 1.65- to compensate the electron loss, resulting in a shoulder peak at 530.5 eV in **Figure 3c**. Next, as mentioned, the Ni^{4+} atom has two empty low-energy orbitals and two empty high-energy orbitals, while the Mn^{4+} atom has 5 low-energy orbitals and 2 high-energy orbitals, causing the empty orbitals ratio is equal to 1:0.470. For the charged M-LLO sample ($Li_{0.1}Ni_{0.6}Mn_{0.6}O_2$), the $Mn^{3.67+}$ atoms are completely oxidized to 4+ during charging based on the result of Mn L-edge sXAS. Nevertheless, the valence of Ni is difficult to identified because the oxidization degree of O atoms is unknown. Here, we first assume that the O atoms are not oxidized, and then the valence of Ni atoms is calculated as 3.75+ by the chemical composition. Hence, the empty orbitals ratio is equal to 1:0.541 according to above discussion. Secondly, the valence of Ni should be lower than 3.75+, leading to the oxidized O atoms. Hence, the valence of Ni is set as the minimum of 2+ and the valence of O atoms can be calculated as 1.65- and the ratio

of orbitals is calculated as 1:0.526, which is also larger than that of P-LLO.

In summary, the ratio of orbitals obtained from the above discussion well illustrate the variation of peak intensity ratios in the O K-edge sXAS curves. The peak intensity at 532 eV is highly related to the valence of TM and Ni content. Since the change of the valence of TM is still induced by the Ni-enrichment structure in M-LLO. Here, we think the enhanced peak intensity at 532 eV for the M-LLO sample is finally ascribed to the Ni-enrichment structure.

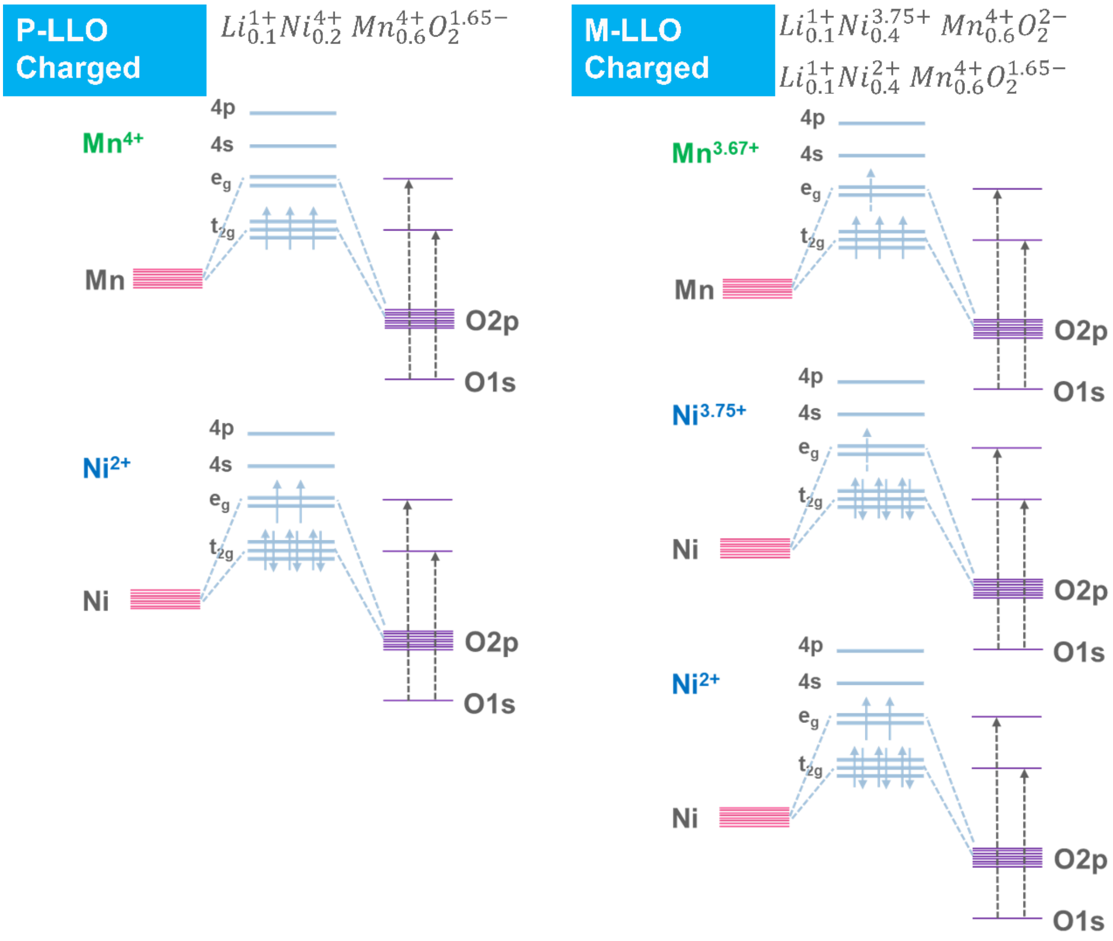

Figure Note2. The electronic arrangement of TM-O hybrid orbitals (e_g and t_{2g}) for the charged P-LLO and M-LLO samples, where Mn is the high-spin system and Ni is the low-spin system.

Table Note2 The atomic ratio, average valence of Ni and Mn atoms, empty orbitals number, and the ratio of orbitals in the charged and uncharged P-LLO and M-LLO samples with 10 nm depth.

Sample	Atomic ratio (Mn:Ni)	Valence		Low-energy orbitals		High-energy orbitals		Orbitals ratio (High:Low)
		Mn	Ni	Mn	Ni	Mn	Ni	
P-LLO (uncharged)	3:1	4+	2+	5	0	2	2	1:0.533
M-LLO (uncharged)	3:2	3.67+	2+	4.67	0	2	2	1:0.713
P-LLO (charged)	3:1	4+	4+	5	2	2	2	1:0.470
M-LLO (charged)	3:2	4+	3.75+	5	1.75	2	2	1:0.541
M-LLO (charged)	3:2	4+	2+	5	2	2	2	1:0.526

References

[1] C. L. Farrow, P. Juhas, J. W. Liu, D. Bryndin, E. S. Božin, J. Bloch, T. Proffen, S. J. L. Billinge, *Journal of Physics: Condensed Matter* **2007**, *19*.

[2] A. Hammersley, *Journal of Applied Crystallography* **2016**, *49*.

[3] a)M. Kraus, W. Wintz, U. Seifert, R. Lipowsky, *Physical Review Letters* **1996**, *77*; b)B. Hammer, L. B. Hansen, J. K. Nørskov, *Physical Review B* **1999**, *59*; c)G. Kresse, J. Furthmüller, *Physical Review B* **1996**, *54*.

[4] a)L. Wang, T. Maxisch, G. Ceder, *Physical Review B* **2006**, *73*; b)A. Jain, S. P. Ong, G. Hautier, W. Chen, W. D. Richards, S. Dacek, S. Cholia, D. Gunter, D. Skinner, G. Ceder, K. A. Persson, *APL Materials* **2013**, *1*.

[5] E. Sanville, S. D. Kenny, R. Smith, G. Henkelman, *J Comput Chem* **2007**, *28*.

[6] V. L. Deringer, A. L. Tchougréeff, R. Dronskowski, *The Journal of Physical Chemistry A* **2011**, *115*.

[7] E. Zhao, M. Zhang, X. Wang, E. Hu, J. Liu, X. Yu, M. Olguin, T. A. Wynn, Y. S. Meng, K. Page, F. Wang, H. Li, X.-Q. Yang, X. Huang, L. Chen, *Energy Storage Materials* **2020**, *24*.

[8] a)Z. Zhu, D. Yu, Y. Yang, C. Su, Y. Huang, Y. Dong, I. Waluyo, B. Wang, A. Hunt, X. Yao, J. Lee, W. Xue, J. Li, *Nature Energy* **2019**, *4*, 1049; b)K. Luo, M. R. Roberts, R. Hao, N. Guerrini, D. M. Pickup, Y.-S. Liu, K. Edström, J. Guo, A. V. Chadwick, L. C. Duda, P. G. Bruce, *Nature Chemistry* **2016**, *8*.
